# Antibiotic Based Phenotype and Hospital Admission Profile are the Most Likely Predictors of Genotyping Classification of MRSA

**DOI:** 10.2174/1874285801711010167

**Published:** 2017-09-21

**Authors:** Ali M. Bazzi, Jaffar A. Al-Tawfiq, Ali A. Rabaan, Dianne Neal, Aimee Ferraro, Mahmoud M. Fawarah

**Affiliations:** 1 Microbiology Lab, Johns Hopkins Aramco Healthcare, Dhahran, Saudi Arabia; 2 Molecular Diagnostic Lab, Johns Hopkins Aramco Healthcare, Dhahran, Saudi Arabia; 3 Specialty Internal Medicine, Johns Hopkins Aramco Healthcare, Dhahran, Saudi Arabia; 4 Indiana University School of Medicine, Indiana, USA; 5 School of Health Sciences, Walden University, Minneapolis, USA

**Keywords:** MRSA, HA-MRSA, CA-MRSA, Saudi Arabia, Genotyping, Phenotyping, Susceptibility Pattern

## Abstract

**Background:**

Methicillin-resistant *Staphylococcus aureus* (MRSA) infection is associated with increased morbidity, mortality, and financial burdens. Phenotyping methods are used to classify MRSA as either health care MRSA (HA-MRSA) or community-associated MRSA (CA-MRSA). Recent studies suggested the phenotyping methods are not always reliable, based on a lack of concordance with genotyping results.

**Objective:**

In this study, concordance of classification methods based on clinical characteristics or antibiotic susceptibility compared to the gold standard genotyping was assessed in the classification of MRSA.

**Methods:**

We compared the genotypes and phenotypes of MRSA in 133 samples taken from patients in Saudi Arabia. Statistical analyses included concordance, specificity and sensitivity, and logistic regression modeling.

**Results:**

There was fair a definite agreement between the health care risk and infection type methods (*p* < .001), but no statistically significant agreement between the susceptibility pattern and health care risk methods (*p* = 243), and between susceptibility pattern and infection type methods (*p* = .919). Reduced multiple regression modelling suggested the potential of a phenotyping-based method of antibiotic susceptibility pattern (OR = 15.47, *p* < .001) in conjunction with hospital admission profile(OR = 2.87, *p* = .008) to accurately identify MRSA as HA-MRSA and CA-MRSA.

**Conclusion:**

The use of a standardized phenotyping technique, using susceptibility pattern and hospital admission profiles to classify MRSA infections as either HA-MRSA or CA-MRSA, would facilitate diagnosis, infection control efforts, prevention, and assignment of appropriate therapies. The ability to use phenotyping in the classification of these strains would improve efforts to contend with this adept and evolving bacterial organism.

## 1 INTRODUCTION

In the early 1960s, Methicillin resistant *Staphylococcus aureus* (MRSA) was first isolated from patients exposed to health care risk factors such as hospitalization, surgery, dialysis, and indwelling devices [1]. Like infections by other multidrug resistant organisms, MRSA infection increases patients’ morbidity and mortality, and health care costs [2]. From the mid-1970s through the 1990s, the incidence of multidrug-resistant *S. aureus*, mainly MRSA, in healthcare settings dramatically increased [3]. Treatment of MRSA infection has been challenging due to the inefficiency of first and second line antibiotics, and use of less effective antibiotics [2].

A nosocomial infection is defined as an infection developing during, or as a result of, an admission to an acute care facility (hospital) and which was not incubating at the time of admission [4]. MRSA is responsible for up to 60% of nosocomial/healthcare associated infections in intensive care units (ICUs); this is likely to be due to carriage of exogenous mobile genetic elements, inappropriate initial antibiotic therapy, and cross transmission between patients [5]. Other risk factors for HA-MRSA include: recent hospitalization, dialysis, residence in a long-term care facility, presence of invasive devices, and history of MRSA infection and colonization [6]. HA-MRSA can cause a variety of diseases from noninvasive infection such as mild abscesses, to life threatening invasive systemic diseases such as necrotizing pneumonia, septic arthritis, and bacteremia [6].

In the late 1990s, community-associated MRSA (CA-MRSA) without healthcare risk factors was identified [1]. Thus, MRSA was categorized into two distinct groups: HA-MRSA and CA-MRSA [6, 7]. CA- MRSA has been described in patients without established healthcare risk factors and was first described in prisoners, intravenous drug users, athletes, military trainees, and men who have sex with men [8, 9].This form of MRSA usually presents as noninvasive infection, such as skin and soft tissue infections.

The CDC reported an encouraging decrease in the rate of HA-MRSA in the United States, with approximately 31,000 fewer cases and 9,000 fewer deaths between 2005 and 2013 [8]. However, in Saudi Arabia, MRSA prevalence in hospitals increased from 5% in 1995 to 35% in 2013 [10].

The epidemiological, biological, and clinical characteristics of MRSA have put pressure on health care settings to apply a variety of phenotyping and genotyping methods for classification of MRSA infections as either HA-MRSA or CA-MRSA [7]. These include three methods as suggested by the CDC: health care risk factor (HCRF), infection type risk factor (ITRF), and antibiotic susceptibility pattern, in addition to genotyping methods. Genotyping methods include Pulse Field Gel Electrophoresis (PFGE), Spa gene sequence typing, Multi Locus Sequence Typing (MLST), or PCR-based detection of the staphylococcal cassette chromosome (*SCCmec*) genes, which is a complex mobile genetic element found in *S. aureus* [11, 12]. In MRSA, it carries the PBP2a-encoding *mec*A gene responsible for the β-lactam antibiotic resistance, that is absent in resistant methicillin-sensitive *Staphylococcus aureus* (MSSA) strains, as well as a cassette chromosome recombinase (ccr) [13, 14]. Classically, HA-MRSA is characterized by a broad antibiotic resistance pattern conferred by carriage of a relatively large SCCmec, usually either SSCmec II or III, which have acquired genes for resistance to antibiotic classes beyond the β-lactams [15]. CA-MRSA, by contrast, is generally associated with the smaller SCCmed types IV and V, and therefore tend to be susceptible to clindamycin and other non-β-lactam antibiotics [16]. CA-MRSA can also be distinguished from HA-MRSA by its expression of the Panton-Valentine toxin (PVL) due to acquisition of prophage-encoded lukS and lukF genes, which encode the dual leukocidin PVL toxin parts, LukS and LukF [17, 18].

According to the HCRF method, HA-MRSA is defined as any MRSA infection identified after 48 hours of hospital admission [7]. According to the ITRF method, HA-MRSA is defined as any MRSA isolated from an invasive site, including blood, cerebrospinal fluid, (CSF), or pleural fluids, from patients who have the following risk factors: hemodialysis, surgery, residence in a long-term care facility or hospitalization during the previous year; or the presence of an indwelling catheter or a percutaneous device at the time of culture. Any MRSA isolated from patients who lack the above risk factors are labeled as CA-MRSA [7].

Based on susceptibility pattern classification, MRSA isolates with resistance only to β-lactams antibiotics are classified as CA-MRSA, whereas resistance to additional antibiotics classes, such as carbapenems, aminoglycoside, and fluoroquinolones, favors HA-MRSA [19]. Such classification is important in monitoring trends in antimicrobial resistance among MRSA within health care settings and for the selection of appropriate antibiotics regimens.

The increase in cases of HA-MRSA and CA-MRSA isolated from health care systems and the communities means that the most effective methods of classification must be available [6]. Although HCRF and ITRF methods are widely used, there has been little examination of their effectiveness or their concordance with the gold standard genotyping. In fact, current classification systems have been challenged in recent studies due to the spread of CA-MRSA into healthcare settings and the emergence of HA-MRSA in the community [20-27]. Previous studies on phenotyping and genotyping concordance suggest that antibiotic susceptibility patterns [19] may be particularly helpful in classification, given the increasingly questioned predictability of healthcare risk factors [20-27]. For example, clindamycin, a macrolide lincosamide-streptogramin B (MLSB) antibiotic, is often used to treat soft tissue and pediatric infections in cases where β-lactams cannot be used. However, inducible clindamycin cross-resistance can arise, usually due to presence of erythromycin ribosomal methylase (erm) encoding genes, resulting in the MLS_B_ inducible phenotype. CA-MRSA strains are usually susceptible to clindamycin, as inducible phenotypes are more common in HA-MRSA due to higher prevalence of the *ermA* gene carried on the transposon Tn554 within SCCmec I, II, and III, but not SCCmec IV. Thus clindamycin susceptibility provides a relatively sensitive and specific classification. However, recent studies have indicated that care is needed in interpretation of results as there is geographic variation in inducible clindamycin resistance, for example in a recent study from Jordan there was a higher rate of inducible clindamycin resistance phenotypes among CA-MRSA compared to HA-MRSA clones [28], in contrast to results of studies in Japan [29-31].

In studies conducted between 1990 and 2013 in the Middle East, and in particular in Saudi Arabia, MRSA was mainly classified based on either the health care risk factor or the susceptibility pattern phenotypic method [32]. One study showed a prevalence of PVL of 30% in a sample of 93 MSSA patients [33]. The absence of a standardized and affordable method to classify MRSA into CA-MRSA and HA-MRSA has been a challenge for infection control programs. A classification scheme appropriate for use at local, regional, and national levels, is needed to harmonize surveillance and treatment programs, in keeping with recommended best practice [34].

The goal of this study was to characterize the MRSA isolates and to improve the classification method used in Saudi Arabia by comparing three classification methods against a nominated gold standard Real Time multiplex PCR method (Qiagen). The PCR method relies on the use of multiple oligonucleotide primers for the simultaneous amplification of several target genes including type I, II, III, IV, and V SCCmec (Table **1**). This methodology identifies SCCmec Types I, II, and III, usually indicating HA-MRSA strains, and Type IV or V genetic element, and PVL genes that usually characterize CA-MRSA [1].

The aim of this study was to answer the following questions (also see Table **2**):

1. What is the distribution of MRSA in Saudi Arabia Eastern Province based on genotyping?
2. What is the concordance between each pair combination of three methods (health care risk factor, infection type, susceptibility pattern) used to classify CA- MRSA vs HA -MRSA in Saudi Arabia?
3. What is the sensitivity and specificity of each method (health care risk factor, infection type, susceptibility pattern) used in Saudi Arabia as compared to the gold-standard used to classify CA- MRSA *vs* HA –MRSA?
4. How well does a combination of demographic and phenotyping variables of the current three methods (health care risk factors, infection type, and susceptibility pattern) predict MRSA genotyping classification as CA-MRSA or HA-MRSA?

## 2 MATERIALS AND METHODS

### 2.1 Study Population

John Hopkins Aramco HealthCare (JHAH) was the primary hub for the collection of samples of patients for this study. The JHAH is a facility with 405 beds, located in Dhahran city in the Eastern Province of Saudi Arabia. JHAH provides medical care for a total population of 350,000.

### 2.2 Data Collection

Secondary data from samples isolated between January 2012 and December 2013 from 157 cases were retrieved from epidemiology and microbiology databases at the JHAH, following formal approval from The Saudi Institutional Review Board. The epidemiological data included the following independent variables: healthcare risk factors, hospital admission profile, whether subjects had been hospitalized for at least 48 hours prior to diagnosis or had been transferred from a different hospital, infection type or bodily location of the infection, and antibiotic susceptibility profile. They also included the covariates age, gender, survival status and pre-existing illnesses.

### 2.3 Classification by Healthcare Risk Factors

Healthcare risk factors classification is currently the methodology used in JHAH for designation of MRSA as HA or CA. Cases had been classified in the database as HA-MRSA if at least one of the following established risk factors was present: hospitalization >48 hours prior to the current infection; presence in an intensive care unit (ICU) >48 hours prior to the current infection; hospitalization in the previous year; surgery during the previous year; dialysis during the previous year; presence of a percutaneous device or indwelling catheter in the previous year; and status as a resident of a long-term care (LTC), nursing home or rehabilitation facility in the previous year [6]. Cases with “no” reported for all seven HA-MRSA risk factors were classified as CA-MRSA [16]. The electronic and/or physical records were re-assessed for the purposes of this study for each sample and the designation as HA-MRSA or CA-MRSA was verified.

### 2.4 Classification by Infection Type using Clinical Information

Cases were classified by the infection type method using the clinical data from the hospital database. A sample was designated as CA-MRSA if a skin or soft tissue infection (SSTI) was diagnosed, including abscess, cellulitis, folliculitis, and impetigo, or if a wound infection had “skin” identified as the culture site. Cases in which more serious infections were detected, including bacteraemia, meningitis, osteomyelitis, pneumonia, septic arthritis, and surgical site infection, were classified as HA-MRSA [6]. If a case had both a SSTI and more invasive infection concurrently, it was considered HA-MRSA [21].

### 2.5 Antibiotic Susceptibility Pattern method using Clinical Information

The operational definition of the antibiotic susceptibly method was according to the CLSI criteria [19]. *S. aureus* samples were primarily tested using an automated microbiology identification system (VITEK-2; bioMerieux, Marcy-l’Etoile, France) system for sensitivity to the following antibiotics: penicillin, oxacillin, gentamicin, ciprofloxacin, levofloxacin, moxifloxacin, clindamycin, erythromycin, quinupristin, linezeloid, vancomycin, and tetracycline. Cefoxitin was also used to determine the presence or absence of MRSA; isolates that were resistant to cefoxitin were assumed to be resistant to oxacillin. VITEK-2 tests for sensitivity were performed by calculating the minimum inhibitory concentration (MIC) for each antibiotic, and the interpretation of each MIC value was assessed based on the CLSI guidelines. Cases were classified as CA-MRSA on the basis of susceptibility patterns if their isolates were resistant only to β-lactams, the basic resistance pattern that defines MRSA (CLSI, 2013). Cases were labelled as HA-MRSA if resistance to additional antimicrobial classes beyond β-lactams was also reported, including but not limited to aminoglycosides, folate pathway inhibitors, lincosinamide, fluoroquinolones, and tetracyclines [16]. 24 samples with MIC to Oxacillin close to the breakpoint of 2 mcg/ml were excluded to avoid any misclassification of borderline oxacillin-resistant *S. aureus* (BORSA) as MRSA.

### 2.6 DNA extraction and Multiplex PCR

MagNA-Pure-Compact-System (Roche Diagnostics, Mannheim, Germany) was used to extract the bacterial DNA according to the manufacturer’s instruction. Briefly, one pure colony of each strain was emulsified in 0.5 ml sterile NaCl (0.9%) and vortexed vigorously. A total of 400 μl of the sample suspension was pipetted into MagNa pure sample tube the manufacturer’s instructions were followed for the DNA-bacterial extraction protocol to give 50 μl total volume.

MRSA strains were classified as either CA-MRSA or HA-MRSA based on the typing analysis by real time PCR of the SCCmec, and detection of PVL-encoding genes (lukS/F-PVL). The primers and probes targeting SCCmec cassettes were designed by TIB Molbiol (Syntheselabor GmbH, Eresburgstr, Berlin) and are listed in Table **1**. PVL primers were luk-PV-1 (5'- ATCATTAGGTAAAA TGTCTGGACATGATCCA-3') and luk-PV-2 (5'- GCATCAA GTGTATTGGATAGCAAAAGC-3') [35].

PCR amplifications were carried out in LightCycler 2.0 instrument. A total of 10 μl volumes containing 2.5 μl of template DNA was used. The PCR conditions were as follows: after 10 min at 95 ^o^C for FastStart Taq DNA polymerase activation, the amplification step was carried for 45 cycles, each with denaturation at 95 ^o^C for 10 s, annealing at 55 ^o^C for 10 s and 12 s elongation at 72 ^o^C. After amplification, a melting curve analysis was completed after 20s denaturation at 95^o^C. Samples were incubated at 40 ^o^C for 20s by continuous heating to 85^o^C with a slope of 0.2^o^C /s.

### 2.7 Data and Statistical Analysis

 SPSS was used to analyze the data. Table **2** shows the four research questions to be answered, and the related study variables and how they were used in statistical analysis including frequency and means, Cohen’s *kappa*, sensitivity and specificity, and logistic regression to answer the four research questions.

To address question 1, descriptive statistics were used, while to address question 2, the bivariate statistic Cohen’s *kappa* was used. In addition to the sensitivy and specificity required for question 3, multivariate logistic regression was used to determine characteristics from phenotyping methods that was most predictive of the genotyping results to address question 4.

## 3 RESULTS

### 3.1 Distribution of MRSA in Saudi Arabia Eastern Province Based on Genotyping

133 distinct MRSA samples were analyzed by multiplex PCR. Of those isolates, 129 (97%) were PVL positive, 61 isolates (46%) had SCCmec II, two (1.5%) isolates had SCCmec III, and 70 (52.5%) isolates had SCCmec IV. Based on this scheme, 47.5% of the MRSA isolates were classified as HA-MRSA and 52.5% as CA-MRSA according to the SCCmec only. This classification did not take into consideration their PVL profile, which was probably influenced by the increased rate of HA-MRSA strains that harbor PVL genes [36-38].

Table **3** shows the distribution of MRSA classified as HA and CA by genotyping compared to the HCRF, ITRF and antibiotic susceptibility methods. 49 of the 63 cases classified as HA-MRSA by genotyping (77.78%) were also identified as HA-MRSA by susceptibility pattern classification. For CA-MRSA identification, 55 out of the 70 cases (78.57%) identified by genotyping were accurately identified by antibiotic susceptibility testing. The proportion of isolates classified as CA-MRSA by genotyping but HA-MRSA by other classification methods differed significantly for the HCRF compared to the susceptibility classification method (22.5% vs 11.2%).

### 3.2 Concordance Between Pairwise Combinations of Three Classification Methods

Concordance or agreement, as shown in Table **4**, on the designation of MRSA type among all three methods was 22% for HA-MRSA, and 16% for CA-MRSA.

### 3.3 Sensitivity and Specificity of Classification Methods

Sensitivity and specificity of each of the three classification methods compared to the genotyping method was used to determine which method best reflects the genotypic identification of strains by PCR. Results are shown in (Tables **5**, **6** and **7**). In this context, sensitivity reflects the ability to identify a case as HA if it is gentoypically HA according to SCCmec PCR, while specificity reflects the ability to identify a case as CA if it is genotypically CA according to SCCmec PCR. The susceptibility method (Table **7**) had higher specificity and sensitivity than either the HCRF method (Table **5**) or the infection type method (Table **6**).

### 3.4 Demographic and Phenotyping Variables that Predict MRSA Genotyping Classification as CA-MRSA or HA-MRSA

To evaluate how well a combination of demographic and phenotyping variables of the current three classification methods predicted results obtained by multiplex PCR-based MRSA genotyping classification, a logistic regression was carried out (Table **8**). Odds Ratios (OR) ranged from 1.01 (.31, 3.84) for the block representing health care risk factor phenotyping method, to 15.474 (5.60, 39.94) for the block representing the susceptibility pattern phenotyping method. Of these blocks, susceptibility pattern was found statistically significant and was confirmed using backwards logistic regression. Of the other independent variables measured in the backwise logistic regression, admission profile was statistically significant with an OR of 3.94 (*p*=.004). These results suggested that a method using admission profile and susceptibility pattern phenotyping would be most efficient in determining whether MRSA is HA or CA in the absence of genotyping methods.

## 4 DISCUSSION

The purpose of this study was to identify which of the three classification methods, health care risk factors, infection type and antibiotic susceptibility pattern, or combination thereof, was best able to classify MRSA strains, with reference to the profile generated using a Multiplex PCR genotyping method. Genotyping methods permit accurate and precise MRSA classification, as markers are less prone to change over time [39]. An additional goal was to help identify the phenotyping variables which were most predictive of genotype and provide an accurate method for development of an effective screening, prevention, control, and treatment program in this population. Accurate phenotyping and the ability to rapidly and efficiently assign MRSA cases to HA-MRSA or CA-MRSA is vital for monitoring trends in MRSA within health care settings and in the community and making choices of appropriate antibiotic treatment, outbreak monitoring, and prediction or recognition of epidemics. Given the rising prevalence of MRSA in Saudi Arabia and the associated rising healthcare costs and increases in morbidity and mortality burdens, these results and the suggestions related to the identification of MRSA strain are essential [40].

Only the susceptibility pattern classification gave a distribution similar to that expected based on genotyping. Neither the healthcare risk factors method nor the infection type method gave a similar distribution to the genotyping method, particularly for CA-MRSA. This is consistent with international experiencein that spread of CA-MRSA into healthcare settings and the emergence of HA-MRSA in the community has challenged assignment of MRSA purely in terms of the healthcare risk and infection type methods [20-27]. It is also consistent with other studies of phenotyping and genotyping concordance, which suggest that antibiotic susceptibility pattern may be particularly helpful in classification, in the context of increasingly questioned predictability of healthcare risk [20-27]. For example, in studies where using a fluoroquinolone susceptibility test, antibiogram results were significantly correlated with genotyping by PFGE, while concordance has also beens shown between the Gene Xpert multiplex PCR genotyping method and antibiotic susceptibility using disk diffusion [43, 44]. The susceptibility pattern method may diverge from the other two methods either because the rate of multidrug resistant CA-MRSA is significantly increased within the JHAH compared to the rate of invasive CA-MRSA, or due to the emergence of invasive CA-MRSA as a nosocomial infection [43].

Remarkably, PVL was detected in 97% of isolates. PVL has been considered to be a marker for distinguishing CA-MRSA from HA-MRSA [17, 18]. However, blurring of the distinctions between CA-MRSA and HA-MRSA challenges this assumption. In an American study, many MRSA infections in patients in hospitals with healthcare risk factors also had many features associated with CA-MRSA, including presence of clindamycin-resistance, PVL positivity, and SCCmec IV [21]. Our recent study on MSSA strains isolated from patients in JHAH showed significant PVL prevalence [33]. Another genotyping study carried out in King Fahad Medical City in Riyadh, Saudi Arabia showed that HA-MRSA resistance markers (e.g., aacA-aphD, aadD) are now common among CA-MRSA strains, while there was a high prevalence of PVL-expressing strains in this healthcare setting [36]. Studies from China have also recently shown high levels of detection of PVL in HA-MRSA strains, with no significant difference in expression levels between CA-MRSA and HA-MRSA samples [37, 38]. Coupled with the results of our current study, it seems that PVL may no longer be a reliable marker of CA-MRSA.

As the goal is to accurately predict the genotyping results for both HA or CA, both a high specificity and a high sensitivity are desirable. According to standard FDA approved techniques, the usual range is 82-100% sensitivity and 64-99% specificity [14]. None of the phenotyping methods used in this study met this standard, however the results for susceptibility pattern suggest a sensitivity of 77.8% (HA identification) and specificity 78.6% (CA identification). All three methods had similar sensitivity, *i.e.* for recognition of HA-MRSA. However, specificity was signficantly lower for the the infection type method in particular, making it significantly less likely than for the other two that CA-MRSA would be identified correctly.

The recommendation based on the results of this study is that a classification method which is built on antibiotic susceptibility pattern and hospital admission data are most likely to predict the genotyping designations of HA-MRSA and CA-MRSA. This is consistent with ecological theory and antibiotics selection pressure theory, which would favor HA-MRSA in a healthcare environment, where antibiotics are commonly used. HA-MRSA usually carries either SSCmec II or III, which have acquired genes for resistance to antibiotic classes beyond the β-lactams [15]. CA-MRSA, on the other hand, tends to carry the relatively smaller SSCmec IV and V, leaving it potentially susceptible to clindamycin and other non-β-lactam antibiotics [16]. However, caution is required, as the classification of ‘HA-MRSA’ and ‘CA-MRSA’ based on SCCmec is also coming under question. MRSA isolates containing SCCmec IV and PVL are arising in the healthcare environment, and SCCmec III has been observed in CA-MRSA strains [21, 38]. Combining antibiotic susceptibility with the admission profile should be helpful in avoiding erroneous classification of CA-MRSA in healthcare settings as HA-MRSA.

The continuous evolution of new MRSA strains has emphasized the need for time and cost saving genotyping method. Proper identification would support MRSA diagnosis and the use of therapies specific to the strain. In this study we designated multiplex PCR as the ‘gold standard’ genotyping method. We recognize that PFGE the generally accepted gold standard MRSA genotyping method as it has high discriminatory power [41, 42, 44, 45]. However, it has various disadvantages that make it an impractical choice in many laboratories, including being technically complicated, time-consuming, and of limited portability, as well as the issue of no major consensus on nomenclature [34, 46, 47]. Other alternatives for genotyping would be spa sequence typing, which has practical advantages including high throughput, rapid turnaround, relatively little technical difficulty, and a standard nomenclature [34]. However, it can lack discrimination in local situations such as that considered in our study [34]. MLST, on the other hand, is highly discriminatory, but is low throughput and expensive [34, 45]. Multiplex PCR is a good compromise method, which offers the advantages of rapidly and relatively easily obtainable results, concordance with phenotypic tests and compatibility with patient management [48, 49].

## CONCLUSION

In conclusion, we have identified antibiotic susceptibility testing in conjunction with hospital admission profile as a potentially valuable method for classification of MRSA in Saudi Arabia.

## Figures and Tables

**Table 1:** SCCmec primers and probes.

**SCCmec Type**	**Primers and Probes Set**
SCCmec I	Forward primer: 5-gTTCTCTCATAgTATgACgTCC-3 Reverse primer: 5-gCTTTAAAgAgTgTCgTTATAgg-3 Oligonucleotidehybridization probes: 5-ATAgCTTTTAAATAATTAAAgATAgggCC--FL LC640-AAgCCTTCAATTgTACCTgAATgTT--PH
SCCmec II	Forward primer: 5-CgAAATCAATggTTAATggACC-3 Reverse primer: 5-CgTTgAAgATgATgAAgCg-3 Oligonucleotidehybridization probes: 5-gCAATTCAgAgACTTTgTggACACACCTAT--FL LC640-TTgTgATTAgTgCACgAACAgAggAACAA--PH
SCCmec III	Forward primer: 5-CCTTAgTTgTCgTAACAgATCg-3 Reverse primer: 5-CCATATTgTgTACgATgCg-3 Oligonucleotidehybridization probes: 5-ACCAACTATACAgTACgTTTgTTAAATCgA--FL LC640-AggACggTCTggTAAAggTTTATTAAgA—PH
SSCmec Iva	Forward primer: 5-gCCTTATTCgAAgAAACCg-3 Reverse primer: 5-CTACTCTTCTgAAAAgCgTCg-3 Oligonucleotidehybridization probes: 5-CTgggAATCTATCACTAATCgCATTATACTT—FL LC640-CCAAAgAATAATAAACATgCTgTAgTCATTTT—PH
SSCmec IVb	Forward primer: 5-AAACAATATTgCTCTCCCTC -3 Reverse primer: 5-TCTggAATTACTTCAgCTgC-3 Oligonucleotidehybridization probes: 5-ATTgTTCAgTTgACCTCTCTTTAATATTTg--FL LC640-CAATACCgCTAAATCTAgTTTTggATACTC--PH
SSCmec IVc	Forward primer: 5-TTggTATgAggTATTgCTgg -3 Reverse primer: 5-ACAATATTTgTATTATCggAgAgC-3 Oligonucleotidehybridization probes: 5-TgTTgAATAATTTTTTCATACgCTTTATCTATT--FL LC640-AATTTTCATCTCTTTCAggTAAATTAgAAACAA--PH
SSCmec IVd	Forward primer: 5-TgCTCCAgTAATTgCTAAAg-3 Reverse primer: 5-CTCAAAATACggACCCCAATACA-3 Oligonucleotidehybridization probes: 5-AAAgCTgAAAAgAAAATACTTgAAgAAATAATgACg--FL LC640-ACTCTCAAATTAAAgAgACAgCAAAAgAgTTAgCAg—PH
SSCmecV	Forward primer: 5- gAACATTgTTACTTAAATgAgCg-3 Reverse primer: 5-TgAAAgTTgTACCCTTgACACC-3 Oligonucleotidehybridization probes: 5-ATCCTCTTCTAATATTTgATTTgAAAATTTgCA--FL LC640-AAATACCATTCTTTAgTCACATCAATgTCATTTgT--PH

**Table 2:** Use of study variables per research question.

**Research Question**	**Variable(s)/ Level of Measurement**	**Statistical Tests**
**1. What is the genotypic distribution of MRSA in a sub-population of Saudi Arabia’s Eastern Province?**	Age/Continuous	Mean and Standard Deviation
	Gender, hospital admission profile, survival, preexisting illnesses, health care risk factors, susceptibility profile(each drug) /Dichotomous	Frequencies
	Infection type/Categorical	Frequencies
**2. What is the concordance between each pair-wise combination of the three phenotyping methods, health care risk factor, infection type, and susceptibility pattern, used to classify CA- vs HA-MRSA in Saudi Arabia?**	Genotype Classification as HA-MRSA and CA-MRSA/ Dependent/Dichotomous	Cohen's Kappa
	Three Phenotyping Methods' Classification as HA-MRSA and CA-MRSA/Independent/ Dichotomous	
**3. What is the sensitivity and specificity of each phenotyping method (health care risk factor, infection type, susceptibility pattern) used to classify CA-MRSA vs HA-MRSA in Saudi Arabia?**	Genotype classification as HA-MRSA and CA-MRSA/ Dichotomous	Sensitivity and specificity
	Three phenotyping methods' classification as HA-MRSA and CA-MRSA/ Dichotomous	
**4. Is it possible to predict HA-MRSA and CA-MRSA in the Eastern Province of Saudi Arabia using a combination of MRSA phenotypical classification factors?**	Health Care Risk Factors	Multiple logistic regression -2 Log Likelihood Hosmer and Lemeshow
	Infection Type	
	Susceptibility Pattern	
	Age/Independent/ Continuous; Gender, hospital admission profile, survival, preexisting illnesses/ Dichotomous	

**Table 3:** Distribution of HA-MRSA and CA-MRSA by genotyping using multiplex PCR.

		**Classification Based on Genotype**		
**Variable**	Values	HA-MRSA (N=63)	CA-MRSA (N=70)	*p* Value
**Gender**	Male	41 (31%)	28 (21%)	.004
	Female	22 (16%)	42 (31.5%)	
**Age**	Mean +/- SD	35.1 +/- 27.3	34.2 +/- 23.9	.839
**Admission Profile**	≥ 48 hours	33 (25%)	20(15%)	.005
	< 48 hours	30 (22.5%)	50 (37.5%)	
**Pre-existing illness**	No	34 (25.5%)	27 (20.3%)	.075
	Yes	29 (21.8%)	43 (32.3%)	
**Health Care risk factors**	HA	42 (31.5%)	30 (22.5%)	.006
	CA	21 (15.8%)	40 (30%)	
**Infection type risk factors**	HA	48 (36%)	44 (33%)	.096
	CA	15 (11.2%)	26 (19.5%)	
**Susceptibility pattern**	HA	49(36.8%)	15(11.2%)	<.001
	CA	14(10.5%)	55(41.3%)	

**Table 4:** Concordance Matrix between non-genotyping classification methods.

	Health Care Risk Factors	Infection Type Risk Factors	Susceptibility Risk Factors	% Cases Matching
**Concordant**	HA	HA	HA	29 (22%)
	CA	CA	CA	22 (16%)

**Table 5:** Sensitivity and specificity of health care risk factors method.

	**MRSA (Genotyping)**	
**Health Care Risk Factor Method**	HA	CA
**HA**	42	30
**CA**	21	40
	Sensitivity = 0.6667	Specificity = 0.5714

**Table 6:** Sensitivity and specificity of infection type method.

	**MRSA (Genotyping)**	
**Infection Type Method**	HA	CA
**HA**	48	44
**CA**	15	26
	Sensitivity = 0.7619	Specificity = 0.3715

**Table 7:** Sensitivity and specificity of susceptibility pattern phenotyping method

Phenotyping (Susceptibility Pattern Method)	MRSA (Genotyping)	
	HA	CA
**HA**	49	15
**CA**	14	55
	Sensitivity = 0.7778	Specificity = 0.7857

**Table 8:** Odds ratios of phenotypical classification factors computed using multivariate binary logistic regression with block entry.

Step	Variable	B	**OR**	95% Confidence Interval	
				**Lower**	**Upper**
**Block 1**	MRSA_HCRF	.100	1.105	.310	3.836
**Block 2**	MRSA_ITRF	.488	1.630	.542	4.898
**Block 3**	MRSA_Susceptibility	2.739	15.474	5.995	39.938
**Block 4**	Gender	.549	1.731	.230	1.450
	Admission Profile	1.056	2.874	.764	10.815
	Pre-existing Illness	.149	1.161	.460	2.932

HCRF: Health Care Risk Factor . ITRF: Infection Risk Factor

## LIST OF ABBREVIATIONS

CA-MRSA: = Community-associated methicillin-resistant *Staphylococcus aureus*
CLSI: = Clinical and Laboratory Standards Institute
CSF: = cerebrospinal fluid
HA-MRSA: = Healthcare-associated methicillin-resistant *Staphylococcus aureus*
HCRF: = Healthcare risk factor
ICUs: = Intensive care units:
ITRF: = Infection type risk factor
JHAH: = John Hopkins Aramco HealthCare
MIC: = minimum inhibitory concentration
MLST: = Multi Locus Sequence Typing
MRSA: = Methicillin-resistant *Staphylococcus aureus*
PFGE: = Pulse Field Gel Electrophoresis
SCCmec: = Staphylococcal cassette chromosome
SSTI: = skin or soft tissue infection

## References

[1] David M.Z., Glikman D., Crawford S.E., Peng J., King K.J., Hostetler M.A., Boyle-Vavra S., Daum R.S. (2008). What is community-associated methicillin-resistant *Staphylococcus aureus?*. J. Infect. Dis..

[2] Centers for Disease Control and Prevention(CDC). (2013). Antibiotic resistance threat in the United States.

[3] Panlilio A.L., Culver D.H., Gaynes R.P., Banerjee S., Henderson T.S., Tolson J.S., Martone W.J. (1992). Methicillin-resistant *Staphylococcus aureus* in U.S. hospitals, 1975-1991.. Infect. Control Hosp. Epidemiol..

[4] Siegel J.D., Rhinehart E., Jackson M., Chiarello L., Healthcare infection control practices advisory committee (2007). Management of multidrug-resistant organisms in health care settings, 2006.. Am. J. Infect. Control.

[5] Inweregbu K., Dave J. (2005). Pittard. A. Nosocomial infections.. Contin. Educ. Anaesth. Crit. Care Pain.

[6] Centers for Disease Control and Prevention (CDC) Methicillin-resistant *Staphylococcus aureus* (2013). (MRSA) Infections..

[7] Centers for Disease Control and Prevention (CDC) (2016). Community associated MRSA information for clinicians..

[8] Kazakova S.V., Hageman J.C., Matava M., Srinivasan A., Phelan L., Garfinkel B., Boo T., McAllister S., Anderson J., Jensen B., Dodson D., Lonsway D., McDougal L.K., Arduino M., Fraser V.J., Killgore G., Tenover F.C., Cody S., Jernigan D.B. (2005). A clone of methicillin-resistant *Staphylococcus aureus* among professional football players.. N. Engl. J. Med..

[9] McCaig L.F., McDonald L.C., Mandal S., Jernigan D.B. (2006). *Staphylococcus aureus*-associated skin and soft tissue infections in ambulatory care.. Emerg. Infect. Dis..

[10] Al-Yousef S., Mahmoud S., Taha M. (2013). Prevalence of methicillin-resistant *staphylococcus aureas* in saudi arabia: Systematic Review and Meta-Analysis.. AJCEM.

[11] Ito T., Kuwahara-Arai K., Katayama Y., Uehara Y., Han X., Kondo Y., Hiramatsu K. (2014). *Staphylococcal* cassette chromosome mec (SCCmec) analysis of MRSA.. Methods Mol. Biol..

[12] Shore A.C., Coleman D.C. (2013). Staphylococcal cassette chromosome mec: recent advances and new insights.. Int. J. Med. Microbiol..

[13] Hiramatsu K., Cui L., Kuroda M., Ito T. (2001). The emergence and evolution of methicillin-resistant *Staphylococcus aureus*.. Trends Microbiol..

[14] Marlowe E.M., Bankowski M.J. (2011). Conventional and molecular methods for the detection of Methicillin-Resistant *staphylococcus aureus*.. J. Clin. Microbiol..

[15] Hiramatsu K., Katayama Y., Yuzawa H., Ito T. (2002). Molecular genetics of methicillin-resistant *Staphylococcus aureus*.. Int. J. Med. Microbiol..

[16] Naimi T.S., LeDell K.H., Como-Sabetti K., Borchardt S.M., Boxrud D.J., Etienne J., Johnson S.K., Vandenesch F., Fridkin S., O’Boyle C., Danila R.N., Lynfield R. (2003). Comparison of community- and health care associated methicillin resistant *Staphylococcus aureus* infection.. JAMA.

[17] Chambers H.F. (2005). Staphylococcal purpura fulminans: A toxin-mediated disease?. Clin. Infect. Dis..

[18] Otto M. (2013). Community-associated MRSA: what makes them special?. Int. J. Med. Microbiol..

[19] Clinical and Laboratory Standards Institute (2013). Performance standards for antimicrobial susceptibility testing. Twenty-third informational supplement. M100-S23. Wayne PA.

[20] Campanile F., Bongiorno D., Falcone M., Vailati F., Pasticci M.B., Perez M., Raglio A., Rumpianesi F., Scuderi C., Suter F., Venditti M., Venturelli C., Ravasio V., Codeluppi M., Stefani S. (2012). Changing Italian nosocomial-community trends and heteroresistance in *Staphylococcus aureus* from bacteremia and endocarditis.. Eur. J. Clin. Microbiol. Infect. Dis..

[21] David M.Z., Glikman D., Crawford S.E., Peng J., King K.J., Hostetler M.A., Boyle-Vavra S., Daum R.S. (2008). What is community-associated methicillin-resistant *Staphylococcus aureus*?. J. Infect. Dis..

[22] Donnio P.Y., Preney L., Gautier-Lerestif A.L., Avril J.L., Lafforgue N. (2004). Changes in staphylococcal cassette chromosome type and antibiotic resistance profile in methicillin-resistant *Staphylococcus aureus* isolates from a French hospital over an 11 year period.. J. Antimicrob. Chemother..

[23] Hetem D.J., Westh H., Boye K., Jarløv J.O., Bonten M.J., Bootsma M.C. (2012). Nosocomial transmission of community-associated methicillin-resistant *Staphylococcus aureus* in Danish Hospitals.. J. Antimicrob. Chemother..

[24] Maree C.L., Daum R.S., Boyle-Vavra S., Matayoshi K., Miller L.G. (2007). Community-associated methicillin-resistant *Staphylococcus aureus* isolates causing healthcare-associated infections.. Emerg. Infect. Dis..

[25] Nair N., Kourbatova E., Poole K., Huckabee C.M., Murray P., Huskins W.C., Blumberg H.M. (2011). Molecular epidemiology of methicillin-resistant *Staphylococcus aureus* (MRSA) among patients admitted to adult intensive care units: the STAR*ICU trial.. Infect. Control Hosp. Epidemiol..

[26] Otter J.A., French G.L. (2011). Community-associated meticillin-resistant *Staphylococcus aureus* strains as a cause of healthcare-associated infection.. J. Hosp. Infect..

[27] Song J.H., Hsueh P.R., Chung D.R., Ko K.S., Kang C.I., Peck K.R., Yeom J.S., Kim S.W., Chang H.H., Kim Y.S., Jung S.I., Son J.S., So T.M., Lalitha M.K., Yang Y., Huang S.G., Wang H., Lu Q., Carlos C.C., Perera J.A., Chiu C.H., Liu J.W., Chongthaleong A., Thamlikitkul V., Van P.H., ANSORP Study Group (2011). Spread of methicillin-resistant *Staphylococcus aureus* between the community and the hospitals in Asian countries: an ANSORP study.. J. Antimicrob. Chemother..

[28] Jarajreh D., Aqel A., Alzoubi H., Al-Zereini W. (2017). Prevalence of inducible clindamycin resistance in methicillin-resistant *Staphylococcus aureus*: the first study in Jordan.. J. Infect. Dev. Ctries..

[29] Otsuka T., Zaraket H., Takano T., Saito K., Dohmae S., Higuchi W., Yamamoto T. (2007). Macrolide-lincosamide-streptogramin B resistance phenotypes and genotypes among *Staphylococcus aureus* clinical isolates in Japan.. Clin. Microbiol. Infect..

[30] Yilmaz G., Aydin K., Iskender S., Caylan R., Koksal I. (2007). Detection and prevalence of inducible clindamycin resistance in *staphylococci*.. J. Med. Microbiol..

[31] Lall M., Sahni A.K. (2014). Prevalence of inducible clindamycin resistance in *Staphylococcus aureus* isolated from clinical samples.. Med. J. Armed Forces India.

[32] Baddour M.M., Abuelkheir M.M., Fatani A.J., Bohol M.F., Al-Ahdal M.N. (2007). Molecular epidemiology of methicillin-resistant *Staphylococcus aureus* (MRSA) isolates from major hospitals in Riyadh, Saudi Arabia.. Can. J. Microbiol..

[33] Bazzi A.M., Rabaan A.A., Fawarah M.M., Al-Tawfiq J.A. (2015). Prevalence of Panton-Valentine leukocidin-positive methicillin-susceptible *Staphylococcus aureus* infections in a Saudi Arabian hospital.. J. Infect. Public Health.

[34] Stefani S., Chung D.R., Lindsay J.A., Friedrich A.W., Kearns A.M., Westh H., Mackenzie F.M. (2012). Meticillin-resistant *Staphylococcus aureus* (MRSA): global epidemiology and harmonisation of typing methods.. Int. J. Antimicrob. Agents.

[35] McClure J.A., Conly J.M., Lau V., Elsayed S., Louie T., Hutchins W., Zhang K. (2006). Novel multiplex PCR assay for detection of the staphylococcal virulence marker Panton-Valentine leukocidin genes and simultaneous discrimination of methicillin-susceptible from resistant *staphylococci*.. J. Clin. Microbiol..

[36] Monecke S., Berger-Bächi B., Coombs G., Holmes A., Kay I., Kearns A., Linde H.J., O’Brien F., Slickers P., Ehricht R. (2007). Comparative genomics and DNA array-based genotyping of pandemic *Staphylococcus aureus* strains encoding Panton-Valentine leukocidin.. Clin. Microbiol. Infect..

[37] Hu Q., Cheng H., Yuan W., Zeng F., Shang W., Tang D., Xue W., Fu J., Zhou R., Zhu J., Yang J., Hu Z., Yuan J., Zhang X., Rao Q., Li S., Chen Z., Hu X., Wu X., Rao X. (2015). Panton-Valentine leukocidin (PVL) positive health care-associated methicillin-resistant *Staphylococcus aureus* isolates are associated with skin and soft tissue infections and colonized mainly by infective PVL encoding bacteriophages.. J. Clin. Microbiol..

[38] Xie X., Bao Y., Ouyang N., Dai X., Pan K., Chen B., Deng Y., Wu X., Xu F., Li H., Huang S. (2016). Molecular epidemiology and characteristic of virulence gene of community-acquired and hospital-acquired methicillin-resistant *Staphylococcus aureus* isolates in Sun Yat-sen Memorial hospital, Guangzhou, Southern China.. BMC Infect. Dis..

[39] Tenover F.C., Arbeit R., Archer G., Biddle J., Byrne S., Goering R., Hancock G., Hébert G.A., Hill B., Hollis R. (1994). Comparison of traditional and molecular methods of typing isolates of *Staphylococcus aureus.*. J. Clin. Microbiol..

[40] Bukharie H.A. (2010). Increasing threat of community-acquired methicillin-resistant *Staphylococcus aureus.*. Am. J. Med. Sci..

[41] Chomvarin C., Tishyadhigama P., Siripornmongcolchai T., Wongwanich S., Limpaiboon T., Chaicumpar K. (2005). Analysis of three phenotyping methods and pulsed-field gel electrophoresis for differentiation of methicillin resistant *Staphylococcus aureus* isolated from two hospitals in Thailand.. Southeast Asian J. Trop. Med. Public Health.

[42] Biendo M., Mammeri H., Pluquet E., Guillon H., Rousseau F., Canarelli B., Belmekki M., Eb F. (2013). Value of Xpert MRSA/SA blood culture assay on the Gene Xpert® Dx System for rapid detection of *Staphylococcus aureus* and coagulase-negative staphylococci in patients with staphylococcal bacteremia.. Diagn. Microbiol. Infect. Dis..

[43] Hudson L.O., Murphy C.R., Spratt B.G., Encenter M.C., Elkins K., Nguyen C., Terpstra L., Gombosev A., Kim D., Hannah P., Mikhail L., Alexander R., Moore D.F., Huang S.S. (2013). Diversity of methicillin-resistant Staphylococcus aureus (MRSA) strains isolated from inpatients of 30 hospitals in Orange County, California.. PLoS One.

[44] Blanc D.S., Struelens M.J., Deplano A., De Ryck R., Hauser P.M., Petignat C., Francioli P. (2001). Epidemiological validation of pulsed-field gel electrophoresis patterns for methicillin-resistant Staphylococcus aureus.. J. Clin. Microbiol..

[45] Deurenberg R.H., Vink C., Kalenic S., Friedrich A.W., Bruggeman C.A., Stobberingh E.E. (2007). The molecular evolution of methicillin-resistant Staphylococcus aureus.. Clin. Microbiol. Infect..

[46] Mehndiratta P.L., Bhalla P. (2012). Typing of Methicillin resistant *Staphylococcus aureus*: a technical review.. Indian J. Med. Microbiol..

[47] Narukawa M., Yasuoka A., Note R., Funada H. (2009). Sequence-based spa typing as a rapid screening method for the areal and nosocomial outbreaks of MRSA.. Tohoku J. Exp. Med..

[48] Malhotra-Kumar S., Haccuria K., Michiels M., Ieven M., Poyart C., Hryniewicz W., Goossens H., MOSAR WP2 Study Team (2008). Current trends in rapid diagnostics for methicillin-resistant Staphylococcus aureus and glycopeptide-resistant enterococcus species.. J. Clin. Microbiol..

[49] Rallapalli S., Verghese S., Verma R.S. (2008). Validation of multiplex PCR strategy for simultaneous detection and identification of methicillin resistant *Staphylococcus aureus.*. Indian J. Med. Microbiol..

